# Supplementary material for: Modelling Skylarks (Alauda arvensis) to Predict Impacts of Changes in Land Management and Policy: Development and Testing of an Agent-Based Model
Source: PLoS One. 2013 Jun 6;8(6):e65803. doi: 10.1371/journal.pone.0065803 (PMC3675089; doi:10.1371/journal.pone.0065803)
Supplement: Supporting Information S4 — The skylark ODdox as a zipped archive. (ZIP) [file pone.0065803.s004.zip › Skylark_ODdox/lowqueue_8h.html]

ALMaSS Skylark ODdox: lowqueue.h File Reference


ALMaSS Skylark ODdox
 2.0


- Main Page
- Related Pages
- Classes
- Files

- File List
- File Members

Classes

lowqueue.h File Reference

**lowqueue.h This file contains the headers for the farm event queue classes**   
More...

`#include <queue>`

|  |  |
| --- | --- |
| Classes | |
| class | LowPriority< ELEMTYPE > |
|  | Used in event handling. More... |
| struct | LowPriPair< ELEMTYPE > |
|  | Used in event handling. More... |

---

## Detailed Description

**lowqueue.h This file contains the headers for the farm event queue classes**

by Frank Nikolaisen   
Version of June 2003   
  
Doxygen formatted comments in July 2008


- CJT
- MSVC
- ALMaSS Working Source
- Landscape
- lowqueue.h
- Generated on Thu Jan 10 2013 13:15:35 for ALMaSS Skylark ODdox by
   1.8.1.1
